# Extracting Knowledge From Scientific Texts on Patient-Derived Cancer Models Using Large Language Models: Algorithm Development and Validation Study

**DOI:** 10.2196/70706

**Published:** 2025-06-30

**Authors:** Jiarui Yao, Zinaida Perova, Tushar Mandloi, Elizabeth Lewis, Helen Parkinson, Guergana Savova

**Affiliations:** 1 Computational Health Informatics Program, Boston Children's Hospital, 401 Park Drive, Boston, MA, United States, 1 7813545014; 2 Harvard Medical School, Boston, MA, United States; 3 European Molecular Biology Laboratory, European Bioinformatics Institute, Hinxton, United Kingdom

**Keywords:** patient-derived cancer models, large language models, knowledge extraction, in-context learning, soft prompting, prompt tuning, information extraction

## Abstract

**Background:**

Patient-derived cancer models (PDCMs) have become essential tools in cancer research and preclinical studies. Consequently, the number of publications on PDCMs has increased significantly over the past decade. Advances in artificial intelligence, particularly in large language models (LLMs), offer promising solutions for extracting knowledge from scientific literature at scale.

**Objective:**

This study aims to investigate LLM-based systems, focusing specifically on prompting techniques for the automated extraction of PDCM-related entities from scientific texts.

**Methods:**

We explore 2 LLM-prompting approaches. The classic method, direct prompting, involves manually designing a prompt. Our direct prompt consists of an instruction, entity-type definitions, gold examples, and a query. In addition, we experiment with a novel and underexplored prompting strategy—soft prompting. Unlike direct prompting, soft prompts are trainable continuous vectors that learn from provided data. We evaluate both prompting approaches across state-of-the-art proprietary and open LLMs.

**Results:**

We manually annotated 100 abstracts of PDCM-relevant papers, focusing on PDCM papers with data deposited in the CancerModels.Org platform. The resulting gold annotations span 15 entity types for a total 3313 entity mentions, which we split across training (2089 entities), development (542 entities) and held-out, eye-off test (682 entities) sets. Evaluation includes the standard metrics of precision or positive predictive value, recall or sensitivity, and *F*_1_-score (harmonic mean of precision and recall) in 2 settings: an exact match setting, where spans of gold and predicted annotations have to match exactly, and an overlapping match setting, where the spans of gold and predicted annotations have to overlap. GPT4-o with direct prompting achieved *F*_1_-scores of 50.48 and 71.36 for exact and overlapping match settings, respectively. In both evaluation settings, LLaMA3 soft prompting improved performance over direct prompting (*F*_1_-score from 7.06 to 46.68 in the exact match setting; and 12.0 to 71.80 in the overlapping evaluation setting). Results with LLaMA3 soft prompting are slightly higher than GPT4-o direct prompting in the overlapping match evaluation setting.

**Conclusions:**

We investigated LLM-prompting techniques for the automatic extraction of PDCM-relevant entities from scientific texts, comparing the traditional direct prompting approach with the emerging soft prompting method. In our experiments, GPT4-o demonstrated strong performance with direct prompting, maintaining competitive results. Meanwhile, soft prompting significantly enhanced the performance of smaller open LLMs. Our findings suggest that training soft prompts on smaller open models can achieve performance levels comparable to those of proprietary very large language models.

## Introduction

Patient-derived cancer models (PDCMs) are created from a patient’s own tumor sample and capture the complexity of human tumors to enable more accurate, personalized drug development and treatment selection. These models, including patient-derived xenografts (PDXs), organoids, and cell lines, allow researchers to test treatments and identify the most effective therapies, and have emerged as indispensable tools in both cancer research and precision medicine. The US National Institutes of Health (NIH) have made significant investments in the generation and characterization of these models, with more than US $3 billion dedicated to active grants referencing PDCMs with a component of their research based on data extracted from the NIH RePORTER [1] for fiscal year 2024 alone. The number of publications using PDCMs continues to increase generating substantial and rich metadata and data that require standardization, harmonization, and integration to maximize the impact of these models and their associated data within the research and clinical communities. CancerModels.Org platform [2] serves as a unified gateway to the largest collection of PDCMs and related data. It empowers researchers and clinicians to discover suitable models for testing research hypotheses, conducting large-scale drug screenings, and advancing precision medicine initiatives. Extraction of PDCM-relevant knowledge and its harmonization within CancerModels.Org is essential to ensure that basic and translational researchers, bioinformaticians, and tool developers have access to PDCM knowledge. While manual curation of publications ensures high accuracy when performed by domain experts, it is time-consuming and labor-intensive. Thus, a more streamlined and efficient knowledge acquisition method is needed to address the growing demand within the scientific community for the timely availability of the PDCM metadata and its associated data.

In parallel, large language models (LLMs) [3-5] often referred to as generative artificial intelligence (AI) systems are trained on vast amounts of data and have demonstrated impressive capabilities in the health care domain [6-8]. Researchers have studied the use of LLMs in addressing various tasks related to health care such as diagnosing conditions [9,10], clinical decision support [11], answering patient questions [12], and medical education [13,14]. It has been shown that LLMs can extract meaningful information from texts [15-17].

In this work, we explore LLM-prompting techniques with the goal of extracting knowledge from PDCM-relevant scholarly publications. We focus on the classic direct prompting [4] and the underexplored soft prompting [18] with state-of-the-art (SOTA) proprietary and open LLMs. Our experimental results provide insights into selecting the optimal prompting methods for specific tasks. The contributions of this paper are:

1. Studying the feasibility of SOTA LLMs as oncology knowledge extractors for PDCM-relevant information from scholarly scientific literature.
2. Creating a manually curated gold dataset spanning 15 entity types for a total 3313 entity mentions from 100 abstracts of PDCM-relevant papers.
3. Researching and comparing, to our knowledge for the first time, direct versus soft prompting techniques for oncology knowledge extraction, specifically PDCM-relevant information from scholarly scientific literature.

## Methods

### Concepts

We define “knowledge” as entities of interest to researchers working with PDCMs in the cancer research field. For example, the patient’s diagnosis provides a reference point to confirm that a PDCM faithfully recapitulates the biology of the original tumor and is essential for ensuring the model’s relevance and reliability in studies of cancer progression or treatment response. Thus, “diagnosis” is important to understand the model’s characteristics in the context of patient’s disease. The patient’s age can significantly affect the molecular and genetic characteristics of the tumor. For example, pediatric cancers often have distinct genetic drivers and tumor microenvironments compared to cancers in older adults. In addition, age-related biological factors, such as immune system, metabolism, and hormone levels, influence how a tumor responds to treatments. Thus, knowing the patient’s age is imperative for predictive accuracy of the model in preclinical testing and relevance of research findings. Therefore, we selected 15 most commonly used CancerModels.Org data model attributes (Table 1), which include the attributes defined in the minimal information standard for patient-derived xenograft models [19] and the draft minimal information standard for in vitro models [20].

**Table 1.** Entity definitions based on the CancerModels.Org data model with examples and interannotator agreement *F*_1_-scores in the exact match setting that requires the spans of the annotators to match exactly.

Entity type	Definition	Example	IAA^a^
diagnosis	Diagnosis at the time of collection of the patient tumor used in the cancer model	TNBC^b^	61.67
age_category	Age category of the patient at the time of tissue sampling	Adult, pediatric	60
genetic_effect	Any form of chromosomal rearrangement or gene-level changes	Missense, amplification	57.67
model_type	Type of patient-derived model	PDX^c^, organoid	53.33
molecular_char	Data or assay generated from or performed on the model in this study	RNA sequencing, whole-exome sequencing	54.33
biomarker	Gene, protein or biological molecule identified in or associated with patient’s/model’s tumor	BRCA1^d^, IDH^e^, epidermal growth factor receptor 2	61.33
treatment	Treatment received by the patient or tested on the model	Surgery, chemotherapy, PARP-inhibitor	55.67
response_to_treatment	Effect of the treatment on the patient’s tumor or model	Progression-free survival, reduced tumor growth	55
sample_type	The type of material used to generate the model or how this material was obtained	Tissue fragment, autopsy	49
tumor_type	Collected tumor type used for generating the model	Primary, recurrent	49.67
cancer_grade	Quantitative or qualitative grade reflecting how quickly the cancer is likely to grow	Grade 1, low-grade	42
cancer_stage	Information about the cancer’s extent in the body according to specific type of cancer staging system	TNM^f^ system, T0, stage I	59.33
clinical_trial	The type of clinical trial or Clinicaltrials.org identifier	Phase II, prospective randomized clinical trials	60.67
host_strain	The name of the mouse host strain where the tissue sample was engrafted for generating the PDX model	NOD-SCID^g^	61.67
model_id	ID of the patient-derived cancer model generated in this study	PHLC402	100

a IAA: interannotator agreement.

b TNBC: triple-negative breast cancer.

c PDX: patient-derived xenograft.

d BRCA1: breast cancer gene 1.

e IDH: isocitrate dehydrogenase.

f TNM: tumor node metastasis.

g NOD-SCID: nonobese diabetic severe combined immunodeficiency.

### Corpus

We used 100 abstracts to develop the gold-standard corpus annotated for the 15 entities (Table 1). The abstracts were chosen from publications linked to the PDCMs submitted to CancerModels.Org platform. They were selected to cover all 3 types of models in the resource-PDXs, organoids, and cell lines. The final corpus is available on GitHub (see Data and Code Availability section).

Three annotators (ZP, TM, and EL) independently labeled entities in all 100 abstracts for a total of 40 hours. The annotation quality was tracked through interannotator agreement (IAA), a measure of agreement between each annotation produced by different annotators working on the same dataset. The IAA is an indication of how difficult the task is for humans and it becomes the target for system development. We used pairwise *F*_1_-score as the IAA metric [21] in the exact match setting that requires the spans of the annotators to match exactly. We computed the agreement between each pair of annotators and averaged across the 3 sets of scores. The final IAA for each entity type is reported in Table 1. The IAA range is 42‐100 indicating moderate agreement. Note that the lowest agreement is for low occurrence entity types, for example, cancer_grade has only 8 instances with 42 IAA. These low-frequency entity types are more likely to be overlooked by the human experts as annotation is a cognitively demanding task. Thus, to ensure a high-quality gold-standard dataset, we overlayed the single annotations with an adjudication step, where the annotators discussed annotation disagreements and potential missed annotations to come to final joint decisions. The resulting gold dataset spans 15 entity types for a total 3313 entity mentions (refer Table 2 for distributions) was split into training, development, and test sets in the standard 60:20:20 ratio. The train set was used for creating entity extraction algorithms, the development set for refining the algorithms, and the test set for the final evaluation.

**Table 2.** Distribution of entity type annotations across training, development, and test sets.

Entity type	Training, n	Development, n	Test, n	Total, n
diagnosis	362	122	114	598
age_category	19	0	0	19
genetic_effect	69	20	33	122
model_type	326	114	110	550
molecular_char	128	37	46	211
biomarker	503	118	163	784
treatment	426	77	130	633
response_to _treatment	99	21	28	148
sample_type	22	8	7	37
tumor_type	61	19	28	108
cancer_grade	6	1	1	8
cancer_stage	7	1	4	12
clinical_trial	35	2	4	41
host_strain	9	0	7	16
model_id	17	2	7	26
Total	2089	542	682	3313

### Prompting Methods

**Figure 1.**
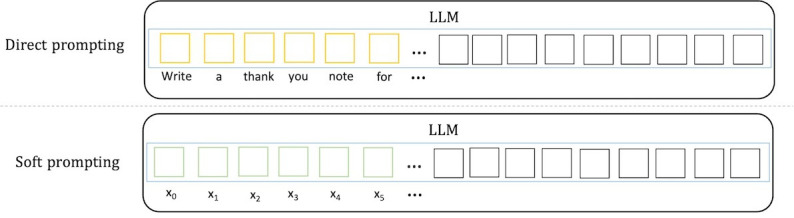
Illustration of the 2 prompting methods. In direct prompting, a prompt contains a sequence of words. In soft prompting, a prompt consists of a list of vectors. LLM: large language model.

Various prompting techniques have been proposed since the emergence of LLMs [22-25]. At a high level, these prompting techniques can be divided into 2 categories, direct prompting [4] and soft prompting [18,24,26] . The main difference between the two methods is the prompt representation, that is whether the prompt consists of human language words or vectors (Figure 1). Direct prompting (or discrete prompting) is the most intuitive and now classic prompting method where users directly interact with LLMs using natural language. For example, a user may ask ChatGPT to “Write a thank you note to an old friend of my parents”; in this case, the text within the quotation marks is a discrete prompt. Soft prompting (or continuous prompting) uses a machine learning approach to train a sequence of continuous vectors, which are the “virtual tokens” of the prompt. It is worth noting that soft prompting differs from fine tuning. With soft prompting, the LLM parameters are not updated, only the soft prompt parameters are adjusted. In contrast, finetuning requires to update the parameters of the entire LLM, and therefore needs more computation resources. Both prompting techniques have their advantages and disadvantages. Compared to direct prompting, soft prompting does not require the tedious process of manually creating prompts; however, it requires some labeled data to train the prompt. In this work, we explore both direct and soft prompting as we aim to explore the latest developments in LLMs and prompting techniques for the task of extracting PDCM entities from abstracts of academic papers.

### Direct Prompting

When asking LLMs to extract entities such as diagnoses or biomarkers, the most intuitive way is to ask LLMs to output the entities directly. In example 1 below, “ALK” is a biomarker entity. One may expect the model to output *{“biomarker” [ALK]}*. However, we note that the string “ALK” is mentioned multiple times in this example text, therefore it is not clear which “ALK” the model refers to. To get the most precise extraction to facilitate a more fine-grained analysis, we instruct the model to output the offsets of the specific mentions in the text (ie, the spans). For instance, if the model gives us *[(48, 51, “ALK,” biomarker), (323, 326, “ALK,” biomarker), …]*, we know that from character 48 to character 51, there is a biomarker entity, “ALK.” Similarly, we can find another biomarker entity “ALK” at position 323‐326.

Example 1:

Oncogenic fusion of anaplastic lymphoma kinase (ALK) with echinoderm microtubule associated protein like 4 protein or other partner genes occurs in 3 to 6% of lung adenocarcinomas. Although fluorescence in situ hybridization (FISH) is the accepted standard for detecting anaplastic lymphoma receptor tyrosine kinase gene (ALK) gene rearrangement that gives rise to new fusion genes, not all ALK FISH-positive patients respond to ALK inhibitor therapies.

We started our exploration by designing prompts with an explicit instruction to specify the character offsets of each entity along with the entity text and type (eg, *48, 51, “ALK”, biomarker*). However, our experiments show that it was challenging for the LLM to output the correct character offsets, a seemingly straightforward task (all the model needs to do is to count the number of characters); however, the complexity of this seemingly straightforward task is likely due to the LLM’s way of breaking words outside its vocabulary into so-called word pieces, for example, “organoid” is broken down into 2 word pieces “organ” and “-oid.” Considering that LLMs were trained as generative models [3,4], we subsequently cast the entity extraction task as a generation task, where we instructed the model to mark the entities with XML tags. For instance, if the model outputs “Oncogenic fusion of anaplastic lymphoma kinase (<biomarker>ALK</biomarker>) with echinoderm microtubule …,” then postprocessing the output with regular expressions would find the exact position of “ALK” in the text. Specifically, we asked the LLMs to mark the start and end of an entity with <entity_type> and </entity_type> tags, where entity_type is a placeholder for the specific entity type, such as biomarker or treatment (refer Table 1 for the full list).

### Soft Prompting

Designing the direct prompts manually could be time-consuming and minor changes in the prompt language could lead to drastic changes in the model performance [24,27]. On the other hand, soft prompting requires some amount of gold data for its training and annotating gold data by domain experts could also be time-consuming. Fortunately, only a small set of labeled data are needed to train soft prompts. As described above, we created a gold dataset, which we used for training and evaluating our soft prompting approach.

There are a few soft prompting methods, the difference usually lies in how the prompt vectors are initialized and learned. Prompt-tuning [18] is a technique that learns the prompt by adding a list of virtual tokens (ie, vectors) in front of the input, where the virtual tokens can be randomly initialized, or drawn from a pretrained word embedding [28] set. Another method is P-tuning [24], which uses small neural networks such as feedforward neural networks [29] (multilayer perceptron) or recurrent neural networks [30] (eg, long-short term memory) as the prompt encoder to learn the prompt. Only the parameters in the prompt encoder are updated during training, while the weights in the LLMs remain frozen. In our experiments, we found P-tuning did not always converge to an optimal solution for our task perhaps due to the random initialization of the vectors rather than using carefully pretrained word embeddings. Therefore, we focused on prompt-tuning in this work. Following Lester et al [18], we initialized the vectors in the prompt with the embeddings of the label words in the entity type set (Table 1).

**Figure 2.**
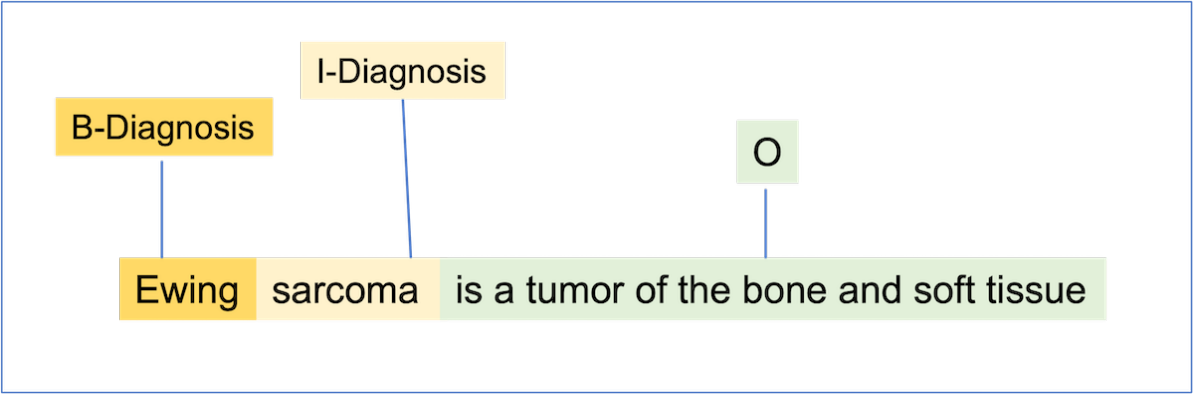
An example of entity extraction as token classification.

The standard approach for entity extraction in natural language processing is via token classification [31]. Concretely, a classifier is trained to predict the label for each token in a sentence according to a predefined label set. Additionally, each label is prepended with a B or I prefix to indicate the entity’s Beginning or Inside mention, respectively. An example is provided in Figure 2. “Ewing sarcoma” is an entity mention of the diagnosis type. Thus “Ewing” and “sarcoma” are labeled as “Diagnosis,” while all other tokens are labeled as “O,” meaning they are Outside of an entity. To be more precise, “Ewing” is at the beginning of the diagnosis entity, and “sarcoma” is inside of the entity, so they are labeled as “B-Diagnosis” and “I**-**Diagnosis,” respectively.

To summarize, we trained a multiclass classifier for the soft-prompting training step. There are 15 entity types in our dataset, therefore there are 15×2+1=31 labels for token classification, with one extra label for “O.”

### Experimental Set-Up

**Figure 3.**
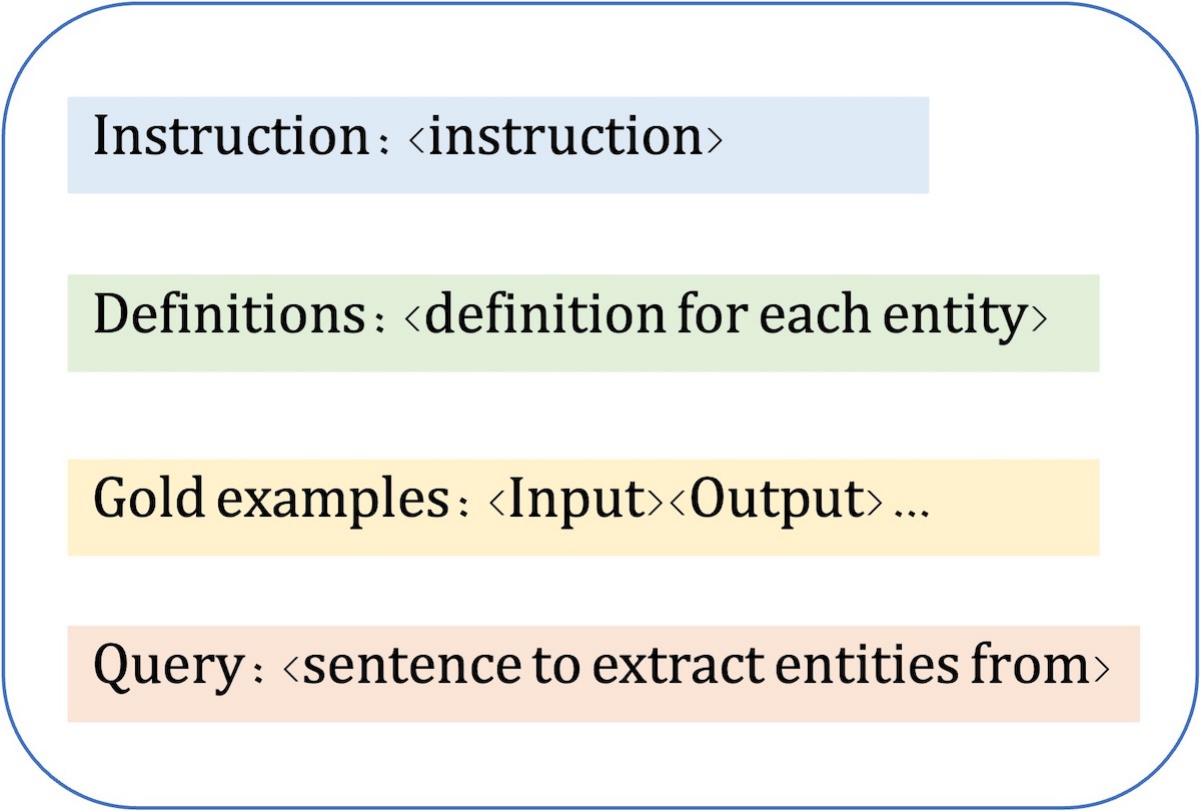
Prompt template used in direct prompting.

For efficiency purposes, we used Apache cTAKES [32] to split an abstract into sentences which were then passed to the LLMs to extract entities one sentence at a time. Our direct prompt included the instruction, the definition of each entity type, 5 examples (few-shot in-context learning) and the query (the sentence). The in-context learning [4] is a common practice in LLM prompting and has consistently shown improved results as the examples guide the LLM onto an optimal path [33,34]. Figure 3 presents our prompt template, and examples are in Multimedia Appendix 1.

When choosing the LLMs, we used GPT-4o [35], one of the most powerful proprietary LLMs at the time of this study, and SOTA open LLMs from the LLaMA3 family [36], including LLaMA3.1 70B, LLaMA3.1 8B, LLaMA3.2 1B, and LLaMA3.2 3B. We did not use GPT-4o or LLaMA3.1 70B to train the soft prompts due to computational resource limitations; thus, our work here is representative of the computational environment in the vast majority of academic medical centers and research labs. We set the soft prompt length to 30. We trained the soft prompt on the training set for 50 epochs with a learning rate of 0.001. Hyperparameters were tuned on the development set using the LLaMA3.1 8B model.

We report the evaluation results on the test set in the next section. In addition, we apply 5-fold cross-validation and report the average scores with SDs. For the 5-fold cross-validation, we excluded the 3 abstracts used to sample the gold examples for direct prompting and split the remaining 97 abstracts into 5 folds with a 20:20:20:20:17 ratio. For direct prompting, we ran the model on each fold and reported the average scores. For soft prompting, we set aside one fold as the test set and trained the soft prompts on the remaining 4 folds.

## Results

We used the standard evaluation metrics of precision or positive predictive value, recall or sensitivity, and *F*_1_-score (the harmonic mean of precision and recall) with 2 evaluation settings: “exact match” setting requires the span output from the model to exactly match the span of the gold annotation, and “overlapping match” setting allows the model to get partial credit if its extraction overlaps the spans in the gold annotation. For example, the model may extract “patient-derived tumor xenograft (PDX)” as a model_type entity, while the gold annotation is “patient-derived tumor xenograft (PDX) models.” Under the “exact match” setting, “patient-derived tumor xenograft (PDX)” is NOT a match to “patient-derived tumor xenograft (PDX) models;” while under the “overlapping match” setting, it is a match since the spans overlap.

Tables3  4 show the evaluation results on the test set. In Table 3, we can see that under the “exact match” setting, GPT-4o direct prompting achieves the highest *F*_1_-score of 50.48. The performances of the LLaMA3 family models drop as the model size decreases, with *F*_1_-score from 38.40 for the 70B model to 6.78 for the 1B model. However, there is a consistent improvement in *F*_1_-scores with soft prompting over direct prompting. For the LLaMA3.2 models, the performance of the 3B model improves significantly, with *F*_1_-score rising from 7.06 to 46.68 *F*_1_-score—more than 8 points higher than the LLaMA3.1-70B model with direct prompting (*F*_1_-score=38.40), despite the substantial difference in model size.

**Table 3.** Evaluation results on the test set (exact match) as precision or positive predictive value, recall or sensitivity, and *F*_1_-score (harmonic mean of precision and recall).

Exact match	Precision	Recall	*F*_1_-score
Direct prompting			
GPT-4o	56.09	45.89^a^	50.48^a^
LLaMA3.1-70B	57.27^a^	28.89	38.40
LLaMA3.1-8B	35.80	18.48	24.37
LLaMA3.2-3B	25.23	4.10	7.06
LLaMA3.2-1B	23.48	3.96	6.78
Soft prompting			
LLaMA3.1-8B	47.17	45.75	46.44
LLaMA3.2-3B	47.30^a^	46.09^a^	46.68^a^
LLaMA3.2-1B	46.19	45.01	45.59

a These are the best results.

**Table 4.** Evaluation results on the test set (overlapping match) as precision or positive predictive value, recall or sensitivity, and *F*_1_-score (harmonic mean of precision and recall).

Overlapping match	Precision	Recall	*F*_1_-score
Direct prompting			
GPT-4o	76.96	66.52^a^	71.36^a^
LLaMA3.1-70B	77.95^a^	43.99	56.24
LLaMA3.1-8B	50.54	27.49	35.61
LLaMA3.2-3B	41.03	7.03	12.00
LLaMA3.2-1B	35.34	6.01	10.28
Soft prompting			
LLaMA3.1-8B	71.19	70.53	70.86
LLaMA3.2-3B	72.05^a^	71.55^a^	71.80^a^
LLaMA3.2-1B	70.38	70.48	70.42

a These are the best results.

Similar trends are observed in Table 4 under the “overlapping match” evaluation. GPT4-o shows an *F*_1_-score performance of 71.36, maintaining its position as the top performer for direct prompting. The 3 smaller LLaMA3 models continue to benefit from soft prompting, with the LLaMA3.2 3B model achieving slightly higher score than GPT4-o with direct prompting (*F*_1_-scores of 71.80 vs 71.36 ).

Tables5  6 present the results with 5-fold cross-validation under “exact match” and “overlapping” match respectively. Once again, our observations indicate that with soft prompting, the smaller LLaMA models attain performance levels comparable to GPT-4o.

**Table 5.** Five-fold cross-validation results (exact match) as precision or positive predictive value, recall or sensitivity, and *F*_1_-score (harmonic mean of precision and recall).

Exact match	Precision	Recall	*F*_1_-score
Direct prompting, mean (SD)			
GPT-4o	60.73 (2.69)	49.92 (3.46)	54.75 (2.84)
LLaMA3.1-70B	57.56 (1.53)	31.70 (1.24)	40.87 (1.25)
LLaMA3.1-8B	38.29 (3.29)	20.57 (2.18)	26.75 (2.61)
LLaMA3.2-3B	27.01 (3.20)	5.25 (0.80)	8.80 (1.29)
LLaMA3.2-1B	9.84 (5.98)	0.74 (0.47)	1.38 (0.87)
Soft prompting, mean (SD)			
LLaMA3.1-8B	51.76 (3.09)	50.21 (2.24)	50.94 (2.55)
LLaMA3.2-3B	50.99 (2.43)	49.54 (2.98)	50.24 (2.53)
LLaMA3.2-1B	49.34 (3.47)	49.98 (3.19)	49.13 (3.10)

**Table 6.** Five-fold cross-validation results (overlapping match) as precision or positive predictive value, recall or sensitivity, and *F*_1_-score (harmonic mean of precision and recall).

Overlapping match	Precision	Recall	*F*_1_-score
Direct prompting, mean (SD)			
GPT-4o	77.82 (2.54)	67.52 (2.17)	72.28 (1.88)
LLaMA3.1-70B	78.01 (1.14)	47.77 (0.71)	59.25 (0.81)
LLaMA3.1-8B	52.75 (3.02)	29.78 (2.60)	38.04 (2.84)
LLaMA3.2-3B	42.42 (2.89)	8.64 (1.09)	14.34 (1.64)
LLaMA3.2-1B	22.09 (5.74)	1.67 (0.54)	3.10 (0.99)
Soft prompting, mean (SD)			
LLaMA3.1-8B	73.78 (3.09)	73.77 (1.25)	73.75 (2.06)
LLaMA3.2-3B	73.48 (1.97)	73.51 (1.11)	73.48 (1.31)
LLaMA3.2-1B	71.51 (3.43)	73.25 (2.46)	72.34 (2.63)

## Discussion

### Principal Findings

Our experiments demonstrate that soft prompting, a relatively underexplored aspect of LLM prompting, can significantly enhance the performance of smaller LLMs. The 3 LLaMA models exhibit comparable performance under soft prompting (an *F*_1_-score of 46 in the exact match setting, and 70 in the overlapping match setting). These results are particularly promising results given the limited training data, consisting of 60 abstracts with 2089 entity mentions. Please note that all *F*_1_-scores mentioned in this section refer to the *F*_1_-scores on the test set.

**Figure 4.**
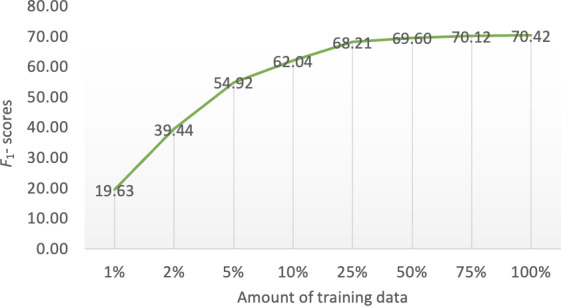
Performance curve of the LLaMA3.2 1B model as the size of training data increases.

How much data is needed to train the soft prompt? To answer this question, we trained LLaMA3.2 1B model, the smallest model used in this work, with different amounts of training data. Figure 4 shows the relation between the proportion of training data and the *F*_1_-scores on the test set (overlapping match). Solid performance was achieved with only 5% of the training data (26 sentences from 3 abstracts). With 25% of the training data (129 sentences from 15 abstracts), the model achieved an *F*_1_-score of 68.21, only 2 points lower than using the entire training set, and only 3 points lower than GPT4-o with direct prompting. Despite the impressive performance of GPT4-o direct prompting, one potential issue is that not all data used in biomedical research can be sent to proprietary models such as GPT or the Gemini family models [8] via public application programming interfaces. That is, for applications using real patient data that require Health Insurance Portability and Accountability Act–compliant platforms, our findings demonstrate that achieving performance comparable to proprietary LLMs such as GPT4-o remains feasible through soft prompting. However, this approach necessitates a tradeoff, requiring a small set of labeled data for optimal effectiveness.

Some entities appear more frequently than other entities in our dataset. For example, diagnosis and treatment mentions are more frequent than mentions of cancer_grade. In Table 7, we present the number of instances of each entity type in our dataset and the corresponding performance of GPT4-o direct prompting. We can see that GPT4-o performs the best for the entity types that have the most instances—diagnosis, model type, and treatment entities. Of these frequent entity types, biomarker is the one with the lowest performance. Our error analysis points to several factors that could have contributed to these results, including ambiguous and inconsistent mentions and contextual dependencies. In this task, we defined a biomarker as “gene, protein or biological molecule identified in or associated with patient’s/model’s tumor.” Thus, biomarker entities can be mentioned using their full names (eg, epidermal growth factor receptor, lnc-RP11-536 K7.3, echinoderm microtubule-associated protein-like 4), standardized gene or protein symbols (*NPM1*, KRAS, PTEN) or abbreviations of metabolites (NADPH, D2HG). Moreover, a biomarker entity (eg, “MEK”) often overlaps with a treatment entity (eg, “MEK inhibitor”). The ambiguity in biomarker entity mentions might interfere with the model’s ability to recognize them consistently. In addition, biomarker entities are often mentioned as lists (see Example 2) resulting in a different frequency within and across the abstracts and patterns of entity mentions, in comparison with other entities. Overall, ambiguity emerges as the primary source of error. More precise definitions, accompanied by examples illustrating the distinct meanings, might present a solution. Table S2 in Multimedia Appendix 1 provides the breakdown of errors per entity type along with examples.

**Table 7.** Evaluation results of GPT4-o with direct prompts for each entity type as precision or positive predictive value, recall or sensitivity, and *F*_1_-score (harmonic mean of precision and recall). Results are overlapping match setting on the test set.

Entity type	Training instances, n	Development instances, n	Test instances, n	Precision	Recall	*F*_1_-score	IAA^a^
diagnosis	362	122	114	92.47	75.44	83.09^b^	61.67
age_category	19	0	0	0.0	0.0	0.0	60
genetic_effect	69	20	33	45.71	47.06	46.38	57.67
model_type	326	114	110	88.07	84.21	86.10^b^	53.33
molecular_char	128	37	46	65.22	63.83	64.52^b^	54.33
biomarker	503	118	163	85.05	55.49	67.16^b^	61.33
treatment	426	77	130	81.74	70.15	75.50^b^	55.67
response_to _treatment	99	21	28	38.64	60.71	47.22	55
sample_type	22	8	7	45.45	71.43	55.56^b^	49
tumor_type	61	19	28	66.67	57.14	61.54^b^	49.67
cancer_grade	6	1	1	50.0	100	66.67^b^	42
cancer_stage	7	1	4	33.33	25.0	28.57	59.33
clinical_trial	35	2	4	80.0	100	88.89^b^	60.67
host_strain	9	0	7	100	28.57	44.44	61.67
model_id	17	2	7	66.67	28.57	40.0	100

a IAA: interannotator agreement.

b *F*_1_-scores exceeding interannotator agreement.

Example 2:

Genomic alterations involved RB1 (55%), TP53 (46%), PTEN (29%), BRCA2 (29%), and AR (27%), and there was a range of androgen receptor signaling and NEPC marker expression.

The moderate performance of entity types such as genetic_effect, molecular_char, and response_to_treatment, and tumour_type is due to the number of training instances ranging from 61 to 128 as well as the IAA ranging from 49.67 to 57.67. The moderate IAA scores of those entity types underscore the need for refined annotation protocols and modeling strategies that better capture domain-specific knowledge. Furthermore, the lower performance observed for entity types with smaller sample sizes (eg, model_id) highlights the need for enhancing model performance on low-frequency labels. Future research could explore strategies such as data augmentation to improve the model’s generalizability for underrepresented entities.

The extraction of PDCM-relevant knowledge is not an easy task for the domain experts as indicated by the IAA (*F*_1_-score below 65 for all entity types except for model_id). In 9 out of 15 entity types, the system performance in an overlapping match setting exceeds the IAA (last two columns of Table 7). This is the case for categories with plentiful training instances (eg, diagnosis, model_type) as well as for categories with fewer training instances (eg, sample_type, cancer_grade). For the exact match setting, in 6 out of 15 entity types, the system performance exceeds the IAA (last two columns in Table 8). Therefore, the LLM could be a viable assistant, with its outputs reviewed by a domain expert to ensure the accuracy of the finalextraction. We believe such human-in-the-loop approaches present a promising direction for future research and application.

**Table 8.** Evaluation results of GPT4-o with direct prompts for each entity type as precision or positive predictive value, recall or sensitivity, and *F*_1_-score (harmonic mean of precision and recall). Results are exact match setting on the test set.

Entity type	Training instances, n	Development instances, n	Test instances, n	Precision	Recall	*F*_1_-score	IAA^a^
diagnosis	362	122	114	77.17	62.28	68.93^b^	61.67
age_category	19	0	0	0.0	0.0	0.0	60.0
genetic_effect	69	20	33	25.71	27.27	26.47	57.67
model_type	326	114	110	56.88	56.36	56.62^b^	53.33
molecular_char	128	37	46	54.35	54.35	54.35^b^	54.33
biomarker	503	118	163	46.74	26.38	33.73	61.33
treatment	426	77	130	72.34	52.31	60.71^b^	55.67
response_to _treatment	99	21	28	27.91	42.86	33.80	55
sample_type	22	8	7	45.45	71.43	55.56^b^	49
tumor_type	61	19	28	50.0	39.29	44.0	49.67
cancer_grade	6	1	1	50.0	100	66.67^b^	42
cancer_stage	7	1	4	33.33	25.0	28.57	59.33
clinical_trial	35	2	4	40.0	50.0	44.44	60.67
host_strain	9	0	7	100	14.29	25.0	61.67
model_id	17	2	7	66.67	28.27	40.0	100

a IAA: interannotator agreement.

b *F*_1_-scores exceeding the interannotator agreement.

We would like to note that the work presented in the paper was done in a computational environment representative of the vast majority of academic medical centers and nonindustry research labs. Although we have access to SOTA Graphics Processing Units, we still found ourselves constrained as to the extent to which we could use very large language models. The larger community needs to address the growing gap in computational resources between big tech and the rest of the research community.

### Limitations

As this is a feasibility study, we limited ourselves to the extraction of entity mentions of 15 entity types chosen from attributes in the descriptive standards for PDCMs. While these are recognized by the PDCM and oncology community, they do not cover all knowledge in the PDCM-relevant texts. Some refinement of the entity types will be beneficial to improve prompting results.

We limited our corpus to 100 abstracts from papers associated with PDCMs deposited in CancerModels.Org. We did not assess the abstracts for the presence and equal distribution of all the entities. Thus, there were very few mentions of some entities in the corpus (eg, cancer_stage), negatively affecting our overall *F*_1_-score. We decided not to exclude those entities as these results could guide refinements of future studies. The computational methods discussed here are applicable to other studies requiring the extraction of textual information from scientific papers. Future work could involve extending this method to extract knowledge from the main body of the papers.

### Conclusions

This study investigates the potential of LLMs as powerful tools for extracting PDCM-relevant knowledge from scientific literature—an essential task for advancing cancer research and precision medicine. By comparing direct and soft prompting across both proprietary and open LLMs, we provide valuable insights into the most effective strategies for PDCM-relevant knowledge extraction. Our findings indicate that GPT-4o, when used with direct prompting, maintains competitive performance, while soft prompting significantly enhances the effectiveness of smaller LLMs. In conclusion, our results demonstrate that training soft prompts on smaller open models can achieve performance levels comparable to those of proprietary LLMs.

To our knowledge, this is the first study to implement SOTA LLMs prompting for knowledge extraction in the PDCM domain and the first to explore the emerging topic of soft prompting in this context. Our findings demonstrate that LLMs can effectively streamline the extraction of complex cancer model metadata, potentially reducing the burden of manual curation and accelerating the integration of PDCMs into research and clinical workflows. Additionally, this study lays the foundation for future research aimed at optimizing LLMs for large-scale knowledge extraction tasks. Efficiently extracting and harmonizing PDCM-relevant knowledge will ultimately drive progress in cancer research and precision oncology, equipping researchers and clinicians with better tools to improve patient outcomes. More broadly, our study contributes to the ongoing discourse on the applicability of LLMs, acknowledging that while they offer transformative potential, they are not a universal solution for all tasks.

## Supplementary material

10.2196/70706 Multimedia Appendix 1 Prompts used in direct prompting experiments and detailed error analysis.

## Abbreviations

IAA: interannotator agreement
LLM: large language model
PDCM: patient-derived cancer model
PDX: patient-derived xenografts
SOTA: state-of-the-art

## References

[1] RePORTER. National Institutes of Health.

[2] Perova Z, Martinez M, Mandloi T (2023). PDCM Finder: an open global research platform for patient-derived cancer models. Nucleic Acids Res.

[3] Vaswani A, Shazeer N, Parmar N (2017). Attention is all you need. arXiv.

[4] Brown TB, Mann B, Ryder N (2020). Language models are few-shot learners. arXiv.

[5] Achiam J, Adler S, OpenAI (2023). GPT-4 technical report. arXiv.

[6] Lee P, Bubeck S, Petro J (2023). Benefits, limits, and risks of GPT-4 as an AI chatbot for medicine. N Engl J Med.

[7] Omiye JA, Gui H, Rezaei SJ, Zou J, Daneshjou R (2024). Large language models in medicine: the potentials and pitfalls: a narrative review. Ann Intern Med.

[8] Saab K, Tu T, Weng WH (2024). Capabilities of Gemini models in medicine. arXiv.

[9] Kanjee Z, Crowe B, Rodman A (2023). Accuracy of a generative artificial intelligence model in a complex diagnostic challenge. JAMA.

[10] Savage T, Nayak A, Gallo R, Rangan E, Chen JH (2024). Diagnostic reasoning prompts reveal the potential for large language model interpretability in medicine. NPJ Digit Med.

[11] Williams CYK, Miao BY, Kornblith AE, Butte AJ (2024). Evaluating the use of large language models to provide clinical recommendations in the emergency department. Nat Commun.

[12] Ayers JW, Poliak A, Dredze M (2023). Comparing physician and artificial intelligence chatbot responses to patient questions posted to a public social media forum. JAMA Intern Med.

[13] Lucas HC, Upperman JS, Robinson JR (2024). A systematic review of large language models and their implications in medical education. Med Educ.

[14] Kung TH, Cheatham M, Medenilla A (2023). Performance of ChatGPT on USMLE: potential for AI-assisted medical education using large language models. PLOS Digit Health.

[15] Perot V, Kang K, Luisier F, Ku LW, Martins A, Srikumar V (2024). Findings of the Association for Computational Linguistics ACL 2024.

[16] Arsenyan V, Bughdaryan S, Shaya F, Small KW, Shahnazaryan D, Demner-Fushman D, Ananiadou S, Miwa M, Roberts K, Tsujii J (2024). Proceedings of the 23rd Workshop on Biomedical Natural Language Processing.

[17] Munnangi M, Feldman S, Wallace B, Amir S, Hope T, Naik A, Duh K, Gomez H, Bethard S (2024). Proceedings of the 2024 Conference of the North American Chapter of the Association for Computational Linguistics.

[18] Lester B, Al-Rfou R, Constant N, Moens MF, Huang X, Specia L, Yih SW (2021). Proceedings of the 2021 Conference on Empirical Methods in Natural Language Processing.

[19] Meehan TF, Conte N, Goldstein T (2017). PDX-MI: minimal information for patient-derived tumor xenograft models. Cancer Res.

[20] PDCMFinder/MI-standard-in-vitro-models. GitHub.

[21] Hripcsak G, Rothschild AS (2005). Agreement, the F-measure, and reliability in information retrieval. J Am Med Inform Assoc.

[22] Wei J, Wang X, Schuurmans D (2022). Chain-of-thought prompting elicits reasoning in large language models. arXiv.

[23] Wang X, Wei J, Schuurmans D (2022). Self-consistency improves chain of thought reasoning in language models. arXiv.

[24] Liu X, Zheng Y, Du Z (2021). GPT understands, too. arXiv.

[25] Schulhoff S, Ilie M, Balepur N (2024). The prompt report: a systematic survey of prompting techniques. arXiv.

[26] Li XL, Liang P (2021). Prefix-tuning: optimizing continuous prompts for generation. arXiv.

[27] Zhou Y, Muresanu A I, Han Z, Paster K, Pitis S, Chan H (2022). Large language models are human-level prompt engineers. arXiv.

[28] Mikolov T, Sutskever I, Chen K, Corrado G, Dean J (2013). Distributed representations of words and phrases and their compositionality. arXiv.

[29] Rosenblatt F (1958). The perceptron: a probabilistic model for information storage and organization in the brain. Psychol Rev.

[30] Hochreiter S, Schmidhuber J (1997). Long short-term memory. Neural Comput.

[31] Tjong Kim Sang EF, De Meulder F Introduction to the CoNLL-2003 shared task: language-independent named entity recognition.

[32] Savova GK, Masanz JJ, Ogren PV (2010). Mayo clinical Text Analysis and Knowledge Extraction System (cTAKES): architecture, component evaluation and applications. J Am Med Inform Assoc.

[33] von Oswald J, Niklasson E, Randazzo E (2022). Transformers learn in-context by gradient descent. arXiv.

[34] Hendel R, Geva M, Globerson A, Bouamor H, Pino J, Bali K (2023). Findings of the Association for Computational Linguistics: EMNLP 2023.

[35] Hurst A, Lerer A, OpenAI (2024). GPT-4o system card. arXiv.

[36] Grattafiori A, Dubey A, Jauhri A (2024). The Llama 3 herd of models. arXiv.

[37] PDCMFinder/prompt-llm. GitHub.

