# Supplementary material for: Extracting Knowledge From Scientific Texts on Patient-Derived Cancer Models Using Large Language Models: Algorithm Development and Validation Study
Source: JMIR Bioinform Biotechnol. 2025 Jun 30;6:e70706. doi: 10.2196/70706 (PMC12232492; doi:10.2196/70706)
Supplement: Multimedia Appendix 1 [file bioinform-v6-e70706-s001.docx]

**Table S1.** Instruction and examples used in direct prompting experiments.

| Instruction | You will be given a sentence from a paper on PDCM. Please extract entities as defined below and return as an XML. In the XML, please mark the start and end of the entity with <entity_type></entity_type>. Please return one entity at one time. That is, if there are n entities in the sentence, print the sentence with the marked entity for n times. Do not change anything else in the sentence. |
| --- | --- |
| Examples | Input:  There were 13 missense mutations identified in the xenograft that were not present in the patient's primary tumor and there were no new nonsense mutations.  Output:  The entities in this sentence are:  {"genetic_effect": [missense mutations], "model_type": [xenograft], "tumour_type": [primary]}  There are 3 entities in total. Now I will print out the sentence for 3 times, each time with only one entity marked, the rest of the sentence will be exactly the same as the original sentence.  (1) There were 13 <genetic_effect>missense mutations</genetic_effect> identified in the xenograft that were not present in the patient's primary tumor and there were no new nonsense mutations.  (2) There were 13 missense mutations identified in the <model_type>xenograft</model_type> that were not present in the patient's primary tumor and there were no new nonsense mutations.  (3) There were 13 missense mutations identified in the xenograft that were not present in the patient's <tumour_type>primary</tumour_type> tumor and there were no new nonsense mutations.  Input:  Here we present the detailed molecular, cellular, genetic and epigenetic characterization of an orthotopic xenograft derived from a high-risk stage 4 neuroblastoma patient.  Output:  The entities in this sentence are:  {"molecular_char": [molecular, cellular, genetic, epigenetic characterization], "model_type": [orthotopic xenograft], "cancer_stage": [stage 4], "diagnosis": [neuroblastoma]}  There are 7 entities in total. Now I will print out the sentence for 7 times, each time with only one entity marked, the rest of the sentence will be exactly the same as the original sentence.  (1) Here we present the detailed <molecular_char>molecular<molecular_char>, cellular, genetic and epigenetic characterization of an orthotopic xenograft derived from a high-risk stage 4 neuroblastoma patient.  (2) Here we present the detailed molecular, <molecular_char>cellular</molecular_char>, genetic and epigenetic characterization of an orthotopic xenograft derived from a high-risk stage 4 neuroblastoma patient.  (3) Here we present the detailed molecular, cellular, <molecular_char>genetic</molecular_char> and epigenetic characterization of an orthotopic xenograft derived from a high-risk stage 4 neuroblastoma patient.  (4) Here we present the detailed molecular, cellular, genetic and <molecular_char>epigenetic characterization</molecular_char> of an orthotopic xenograft derived from a high-risk stage 4 neuroblastoma patient.  (5) Here we present the detailed molecular, cellular, genetic and epigenetic characterization of an <model_type>orthotopic xenograft</model_type> derived from a high-risk stage 4 neuroblastoma patient.  (6) Here we present the detailed molecular, cellular, genetic and epigenetic characterization of an orthotopic xenograft derived from a high-risk <cancer_stage>stage 4</cancer_stage> neuroblastoma patient.  (7) Here we present the detailed molecular, cellular, genetic and epigenetic characterization of an orthotopic xenograft derived from a high-risk stage 4 <diagnosis>neuroblastoma</diagnosis> patient.  Input:  Neuroblastoma is a pediatric cancer of the developing sympathoadrenal lineage.  Output:  The entities in this sentence are:  {"diagnosis": [Neuroblastoma], "age_category": [pediatric]}  There are 2 entities in total. Now I will print out the sentence for 2 times, each time with only one entity marked, the rest of the sentence will be exactly the same as the original sentence.  (1) <diagnosis>Neuroblastoma</diagnosis> is a pediatric cancer of the developing sympathoadrenal lineage.  (2) Neuroblastoma is a <age_category>pediatric</age_category> cancer of the developing sympathoadrenal lineage.  Input:  With these PDXs, we performed in vivo compound screens using a 1 × 1 × 1 experimental design (PDX clinical trial or PCT) to assess the population responses to 62 treatments across six indications.  Output:  The entities in this sentence are:  {"model_type": [PDXs], "clinical_trial": [PDX clinical trial, PCT]}  There are 3 entities in total. Now I will print out the sentence for 3 times, each time with only one entity marked, the rest of the sentence will be exactly the same as the original sentence.  (1) With these <model_type>PDXs<model_type>, we performed in vivo compound screens using a 1 × 1 × 1 experimental design (PDX clinical trial or PCT) to assess the population responses to 62 treatments across six indications.  (2) With these PDXs, we performed in vivo compound screens using a 1 × 1 × 1 experimental design (<clinical_trial>PDX clinical trial</clinical_trial> or PCT) to assess the population responses to 62 treatments across six indications.  (3) With these PDXs, we performed in vivo compound screens using a 1 × 1 × 1 experimental design (PDX clinical trial or <clinical_trial>PCT<clinical_trial>) to assess the population responses to 62 treatments across six indications.  Input:  As autopsy specimens had an ALK R1181C mutation, PDX tumor bearing animals were treated with the pan-kinase inhibitor lestaurtinib but demonstrated no decrease in tumor growth.  Output:  The entities in this sentence are:  {"sample_type": [autopsy], "biomarker": [ALK], "model_type": [PDX], "treatment": [pan-kinase inhibitor, lestaurtinib], "response_to_treatment": [decrease in tumor growth]}  There are 6 entities in total. Now I will print out the sentence for 6 times, each time with only one entity marked, the rest of the sentence will be exactly the same as the original sentence.  (1) As <sample_type>autopsy</sample_type> specimens had an ALK R1181C mutation, PDX tumor bearing animals were treated with the pan-kinase inhibitor lestaurtinib but demonstrated no decrease in tumor growth.  (2) As autopsy specimens had an <biomarker>ALK<biomarker> R1181C mutation, PDX tumor bearing animals were treated with the pan-kinase inhibitor lestaurtinib but demonstrated no decrease in tumor growth.  (3) As autopsy specimens had an ALK R1181C mutation, <model_type>PDX</model_type> tumor bearing animals were treated with the pan-kinase inhibitor lestaurtinib but demonstrated no decrease in tumor growth.  (4) As autopsy specimens had an ALK R1181C mutation, PDX tumor bearing animals were treated with the <treatment>pan-kinase inhibitor</treatment> lestaurtinib but demonstrated no decrease in tumor growth.  (5) As autopsy specimens had an ALK R1181C mutation, PDX tumor bearing animals were treated with the pan-kinase inhibitor <treatment>lestaurtinib</treatment> but demonstrated no decrease in tumor growth.  (6) As autopsy specimens had an ALK R1181C mutation, PDX tumor bearing animals were treated with the pan-kinase inhibitor lestaurtinib but demonstrated no <response_to_treatment>decrease in tumor growth</response_to_treatment>. |

**Table S2.** Summary of GPT-4o errors (overlapping match) on the test set.

| Entity Types | # False Negative (FN) | FN Examples | # False Positive (FP) | FP Examples |
| --- | --- | --- | --- | --- |
| diagnosis | 28 | “BRCA1-methylated breast cancers”, “NEPC” | 7 | “breast”, “small-cell neuroendocrine morphology” |
| genetic_effect | 18 | “deficient”, “methylated” | 19 | “mutated gene”,  “genomic alterations” |
| model_type | 18 | “3D organoids”, “isogenic cell lines” | 13 | “models”,  “preclinical models” |
| molecular_char | 17 | “somatic copy number alteration”, “TLA” | 16 | “immunohistochemistry”, “molecular” |
| biomarker | 73 | “FGFR1/2/3”, “Aurora A/B”, “Aurora A or B kinase” | 16 | “predictive biomarker”, “OXPHOS dependency”, “neuroendocrine markers” |
| treatment | 40 | “tinengotinib”, “TKI”, “EGFR TKI” | 21 | “targeted therapies”, “chemotherapeutic” |
| response_to_treatment | 11 | “antitumor activity”, “inhibited proliferation” | 27 | “do not respond”,  “high response rates” |
| sample_type | 2 | “surgical resection”, “serous effusions” | 6 | “normal tissue”, “tumor tissue” |
| tumour_type | 12 | “talazoparib sensitive”, “metastatic” | 8 | “treatment resistant”, “metastasis-derived” |
| cancer_grade | 0 | N/A | 1 | “residual” |
| cancer_stage | 3 | “advanced” | 2 | “advanced” |
| clinical_trial | 0 | N/A | 1 | “first-in-human” |
| host_strain | 5 | “NOD-SCID”, “nonobese diabetic severe combined immune deficient” | 0 | N/A |
| model_id | 5 | “COH-SC31” | 1 | “HCC1806” |
